# The Heroic Conduct of the Surgeon of the “Tayleur”

**Published:** 1854-04

**Authors:** 


The Heroic Conduct of the Surgeon of the "Tayleur."—Most of our readers have probably read in the newspapers an account of the heroic conduct of Mr. Cunningham, the surgeon of the ship *Tayleur*, recently wrecked on the coast of England. The following just and beautiful tribute to his memory is from the pen of Dickens:—

"When these men perish at their work, they do not die with soldiers' laurels, but their names become connected with their last brave actions, and are told by Englishmen to one another in their households, so that in after years they receive honor by many a fireside. The surgeon of the *Tayleur* was conspicuous in his exertions for the re-assurance and assistance of the shipwrecked passengers. We read at home, how, while struggling across a rope, with his own infant in his hands and teeth, he was plunged into the sea that dashed his child out of his hold; we read that he was seen, then, holding by the ship's side with a drowning woman in his arms, whose hair he was parting gently, and to whom he seemed to be speaking words of comfort. Her, too, the sea forced from his grasp; and we read that he was next seen perishing with his wife, during a vain struggle to save her. The noble man with his little family—his wife and two children—is swept away; he exists now only in the name of Robert Hannay Cunningham. But these are the men whom we want living among us; these are the energies that we need for the leavening of all society, and for the work of the world. These are not men to be sent out in emigrant ships to the bottom of the sea. Their memory, too, will be best honored if we be indignantly aroused, for their sakes, to amend an evil."—*Lancet.*

